# Cellular senescence in the pathogenesis of pulmonary arterial hypertension: the good, the bad and the uncertain

**DOI:** 10.3389/fimmu.2024.1403669

**Published:** 2024-08-02

**Authors:** Elmira Safaie Qamsari, Duncan J. Stewart

**Affiliations:** ^1^ Sinclair Centre for Regenerative Medicine Program, Ottawa Hospital Research Institute, Ottawa, ON, Canada; ^2^ Department of Cellular and Molecular Medicine, Faculty of Medicine, University of Ottawa, Ottawa, ON, Canada; ^3^ Division of Cardiology, University of Ottawa Heart Institute, Ottawa, ON, Canada

**Keywords:** endothelial cells, senescence, PAH, pulmonary hypertension, senotherapeutic, pulmonary arterial hypertension

## Abstract

Senescence refers to a cellular state marked by irreversible cell cycle arrest and the secretion of pro-inflammatory and tissue-remodeling factors. The senescence associated secretory phenotype (SASP) impacts the tissue microenvironment and provides cues for the immune system to eliminate senescent cells (SCs). Cellular senescence has a dual nature; it can be beneficial during embryonic development, tissue repair, and tumor suppression, but it can also be detrimental in the context of chronic stress, persistent tissue injury, together with an impairment in SC clearance. Recently, the accumulation of SCs has been implicated in the pathogenesis of pulmonary arterial hypertension (PAH), a progressive condition affecting the pre-capillary pulmonary arterial bed. PAH is characterized by endothelial cell (EC) injury, inflammation, and proliferative arterial remodeling, which leads to right heart failure and premature mortality. While vasodilator therapies can improve symptoms, there are currently no approved treatments capable of reversing the obliterative arterial remodeling. Ongoing endothelial injury and dysfunction is central to the development of PAH, perpetuated by hemodynamic perturbation leading to pathological intimal shear stress. The precise role of senescent ECs in PAH remains unclear. Cellular senescence may facilitate endothelial repair, particularly in the early stages of disease. However, in more advanced disease the accumulation of senescent ECs may promote vascular inflammation and occlusive arterial remodeling. In this review, we will examine the evidence that supports a role of endothelial cell senescence to the pathogenesis of PAH. Furthermore, we will compare and discuss the apparent contradictory outcomes with the use of interventions targeting cellular senescence in the context of experimental models of pulmonary hypertension. Finally, we will attempt to propose a framework for the understanding of the complex interplay between EC injury, senescence, inflammation and arterial remodeling, which can guide further research in this area and the development of effective therapeutic strategies.

## 1 Introduction

Pulmonary arterial hypertension (PAH) is a rare lung vascular disease characterized by complex arterial remodeling that leads to narrowing and obliteration of the distal lung arterial bed, progressive increases in pulmonary arterial pressure and resistance, and ultimately right heart failure and death (1). PAH is triggered by endothelial cell (EC) injury and apoptosis (2). EC apoptosis and loss can result directly in distal arteriolar pruning and indirectly induces reactive EC proliferation to repair and regenerate the damaged lung microcirculation (3–5). With ongoing endothelial stress, an imbalance between endothelial injury and repair likely sets the stage for maladaptive arterial remodeling and vascular inflammation which are both typical pathological features of PAH (2). Senescence is a cellular state characterized by irreversible cell cycle arrest and the secretion of a pro-inflammatory and tissue-remodeling factors called the senescence-associated secretory phenotype (SASP) (6, 7). Accumulation of senescent cells can lead to altered inflammatory state and cellular senescence has been recognized as a promising target in the treatment of a number of pulmonary and cardiovascular diseases (8, 9). In PAH, senescent cells, including smooth muscle and ECs, are mainly localized to remodeled arteries and have been implicated in the progression from reversible to irreversible disease (10). However, interventions targeting cellular senescence in PAH have produced conflicting results (10–12), either greatly improving arterial remodeling and pulmonary hemodynamics or exacerbating the progression of disease, depending on the models studied and the timing of the intervention.

In this review we will explore the possible role of EC senescence in lung microvascular repair at early stages of PAH as well as its contribution in promoting vascular inflammation and remodeling in more advanced disease. We will also address the potential pitfalls in the use of senotherapeutic agents and discuss alternative strategies to target the accumulation of senescent cells in PAH. Further exploration of the role of senescence in the pathobiology of PAH may point to new novel treatment strategies to target cellular senescence, with the goal of reducing PAH progression, mortality, and reversing the disease.

## 2 Pulmonary hypertension

Pulmonary hypertension (PH) represents a range of heterogeneous conditions which exhibit elevated pulmonary artery pressure (mean > 20 mmHg) and are classified into five separate groups based underlying etiology and clinical management approaches (13). Pulmonary arterial hypertension (PAH), or group 1 pulmonary hypertension (PH), is a rare and often fatal condition resulting from narrowing or loss of the precapillary arterial bed as defined hemodynamically by a normal pulmonary wedge pressure (mean < 15 mmHg) and high pulmonary vascular resistance (mean > 3 Wood units) (14). There are a number of clinical subtypes of PAH, including: idiopathic PAH; PAH associated with another disease, typically connective tissue diseases and congenital heart diseases (CHD) with large systemic to pulmonary arterial shunts; and heritable (H) PAH, which is most commonly caused by mutations in the bone morphogenetic protein receptor type II (*Bmpr2*) gene. While the pathogenesis of PAH is complex, it is generally accepted that EC injury and/or apoptosis caused by toxins, inflammation, or other noxious stimuli represents a triggering mechanism in the context of a genetic or other predisposition. This can result in EC dysfunction, increased vasoconstriction and ultimately pulmonary arterial remodeling and inflammation affecting both larger arteries and small arterioles (2), including the development of complex arterial remodeling, which is a hallmark feature of PAH (15). Although endothelial dysfunction and simple medial arterial thickening have been seen in all forms of PH, PAH is characterized by intimal lesions resulting in luminal encroachment and occlusion and often associated with adventitial remodeling and inflammation. The resulting complex arterial remodeling has been variously termed, ‘plexiform’ or ‘angioproliferative’, and is usually seen at branching points of the pulmonary arteries (16). Moreover, over the last three decades, a number of PAH-specific treatments have been approved, all of which address endothelial dysfunction and vasoconstriction rather than complex arterial remodeling (17). While these vasodilator agents can improve functional capacity, pulmonary hemodynamics and reduce hospitalization (18), their effect on survival is less clear of despite all available therapy, PAH, still carries a poor outlook, eventually leading to right heart failure and death with a 5-year survival of little more than 50% in a recent study (17), underlining the need for novel therapeutic approaches. Although several studies have elucidated the importance of endothelial senescence in atherosclerosis, heart failure, and metabolic disorders, its role in the pathogenesis of PAH is unclear (19). Consequently, gaining insight into the role of endothelial senescence in this disease may pave the way for innovative therapeutic approaches for the treatment of this otherwise progressive and often lethal condition.

## 3 Cellular senescence

Cellular senescence refers to a state of permanent growth arrest induced by stress as was initially described in cultured fibroblasts (15) Unfortunately, there is no single marker for identifying senescent cells (SCs). A SC is typically characterized by its enlarged, flattened appearance, vacuolized cellular morphology, elevated cytoplasm-to-nucleus ratio, and occasionally, the presence of multiple nuclei (6). These cells exhibit high expression of cyclin-dependent kinase inhibitors, such as p21Cip1 and p16INK4a, and the accumulation of the lysosomal enzyme senescence-associated-β-galactosidase (SA β-gal). SCs characteristically secrete a variety of inflammatory cytokines, chemokines, growth factors and proteases, termed senescence-associated secretory phenotype (SASP), which can contribute to a proinflammatory micro-environment (7). Senescence reprogramming is not a static cell fate; rather, it is a dynamic, multi-step process characterized by distinct molecular signatures determined by heterochromatin rearrangement and metabolic changes (15). Cellular senescence can be induced by a wide variety of noxious stimuli, including persistent DNA damage, oxidative stress, mitochondrial dysfunction, oncogene activation (leading to oncogene-induced senescence), and telomere shortening (7).

**Figure 1:**
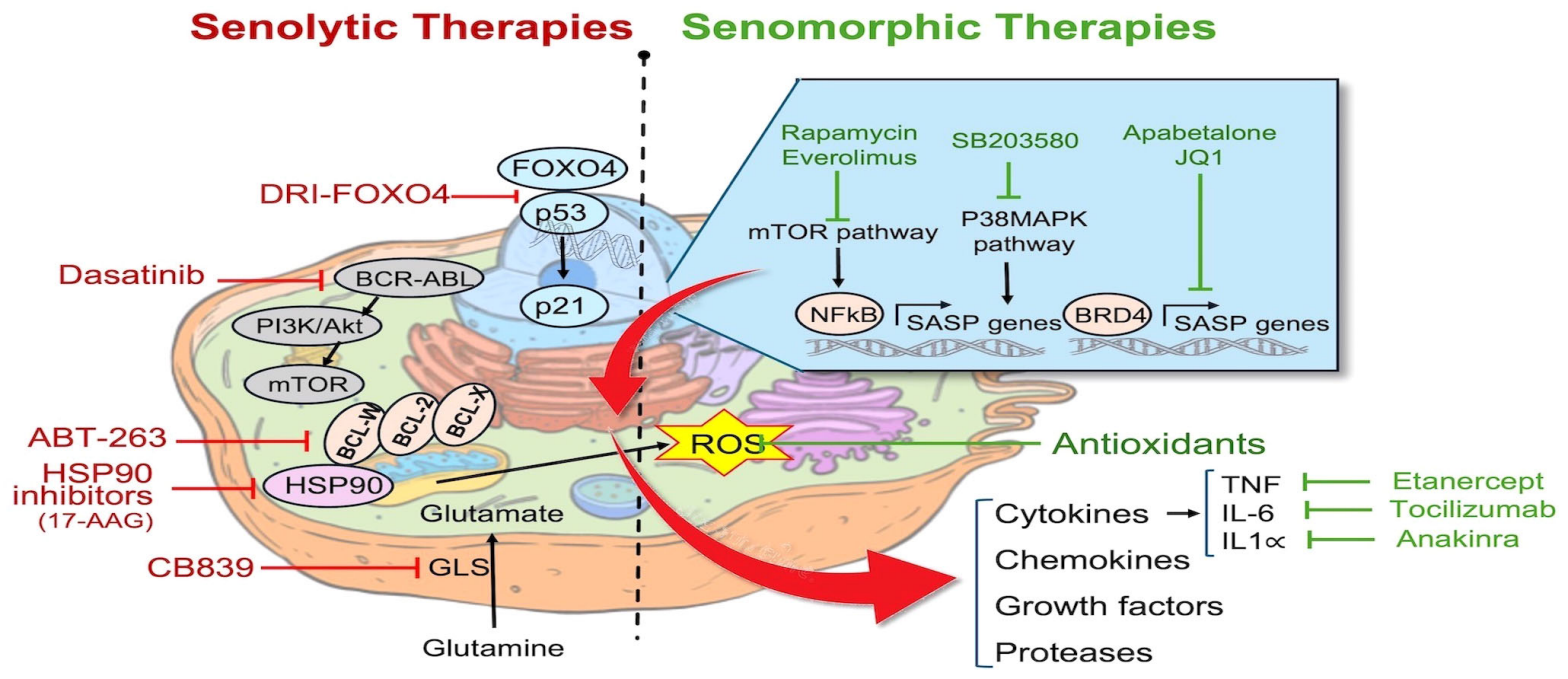
Pathways targeted by senotherapies: Senotherapies comprise agents aimed at eliminating senescent cells by either inducing apoptosis (senolytics) or attenuating inflammation caused by the SASP (senomorphics). Examples of senolytics agents include FOXO4-interacting peptide (DIR-FOXO4), BCL2 family inhibitors (ABT-263), HSP90 chaperone inhibitors, tyrosine kinase inhibitor (Dasatinib), and Glutaminase inhibitors (CB839). Senomorphic agents include mTOR inhibitors (Rapamycin, Everolimus), p38 MAPK inhibitors (SB203580), BET protein (BRD4) inhibitors (Apabetalone, JQ1), antioxidants, and inhibitors of specific cytokines including Etanercept, Tocilizumab, and Anakinra. Cell structure template modified from: https://timvandevall.com/animal-cell-diagram/.

Cellular senescence is not always pathological, and it has recently been recognized to be adaptive in a range of physiological processes, including embryonic development, cellular reprogramming, and responses to tissue injury, such as wound healing and tissue repair. Exiting the cell cycle, as opposed to undergoing apoptosis, offers physiological advantages, and the release of chemokines as part the SASP, such as colony stimulating factor 1 (CSF1), CCL2 and IL-8, serves as a signal for immunosurveillance to promote self-clearance of senescent cells and prevent a chronic inflammatory response (20). In contrast, senescent phenotype can also play an important role in age-related diseases, including chronic cardiovascular and lung diseases, such as atherosclerosis (21) and pulmonary fibrosis (15, 22). Indeed, the accumulation of SCs in tissues can hinder repair while, at the same time, foster both paracrine and endocrine inflammation through release of the bioactive SASP (19). A three-step multi-marker method has been suggested to enhance the precision of identifying SCs involving: 1) the initial screening for senescence using markers (e.g., SA-β-gal or lipofuscin accumulation); 2) subsequent co-staining for the expression of other genes commonly found in SCs (e.g., P16, P21) or the absence of proliferation markers (e.g. Ki67 and Lamin B1); and 3) the identification of factors associated cellular senescence in a specific context, including the presence of SASP, evidence of DNA damage and senescence associated-signaling pathways such as PI3K/FOXO/mTOR as illustrated in **Figure 1** (23). Importantly, expression of each characteristic of cellular senescence depends on the context, and these can differ depending on the nature of the stress trigger, the specific cell or tissue type, and the time elapsed since the initiation of the senescence program (24).

## 4 Endothelial cell senescence

The endothelium is a dynamic cell monolayer representing the inner lining all blood vessels and playing an essential role in vascular development and the control of vascular tone and structure, while providing a thromboresistant surface to maintain blood fluidity and selective vascular permeability (19). The endothelium is highly heterogeneous, with profound differences in its structure and function in different tissue beds and in different regions of the vasculature (25). ECs are continuously exposed to shear forces related to blood flow and a wide variety of circulating factors, both physiological and pathological. Under normal conditions, the release of endothelial factors such as NO, plays a vital role in maintaining appropriate vascular tone and inhibiting platelet adhesion and activation (26). However, in pathological states, the endothelium is often the initial target for vascular injury (19), resulting in impaired tissue and organ function by disrupting blood flow and barrier function. Additionally, ‘activated’ ECs can release vasoconstrictor factors, such as endothelin-1 (27), and pro-inflammatory cytokines and chemokines (28) which can transmit damaging signals to other vascular and non-vascular cells and tissues. Sustained endothelial damage can also result in EC senescence, which has been implicated in a wide variety of vascular diseases (15). Endothelial senescence has been observed in different vascular regions, including the kidney, retina, liver, brain, lung, and aorta, consistent with a role in numerous pathological processes linked to vascular dysfunction (19). Several studies have emphasized the significance of endothelial senescence, especially in the context of pulmonary diseases. Amsellem et al. reported an elevated percentage of senescent lung ECs in individuals with chronic obstructive pulmonary disease, driven by telomere erosion and associated with increased inflammatory signaling mediated by IL-6, IL-8, MCP1 (monocyte chemotactic protein 1), and sICAM-1 (soluble intercellular adhesion molecule 1) (29). Senescent ECs have also been shown to play a detrimental role in the progression of pulmonary fibrosis (30), potentially by promoting endothelial to mesenchymal transition (EndMT) and enhancing the myofibroblastic transition of resident lung fibroblasts. This study suggested that targeting senescent ECs could be a promising pharmacotherapeutic approach for preventing and treating idiopathic pulmonary fibrosis (IPF), particularly in the elderly population. As well, it was recently reported that cellular senescence plays a role in the progression of bronchopulmonary dysplasia (BPD) (31), a disease of arrested lung development in extremely premature infants (32). This was evidenced by an increase in cellular senescence markers in the type-2 alveolar epithelial cells (AT2) and ECs in both a rat model and human BPD lungs, including elevated levels of oxidative DNA damage, tumor suppressors, GL-13, and inflammatory cytokines, along with reduced cell proliferation and lamin B expression. Administration of DRI-FOXO4, a senolytic agent that antagonizes Foxo4 binding to p53, improved lung development, suggesting that this may be a novel treatment strategy for BPD (31, 33). Therefore, as in these conditions the release of inflammatory cytokines and part of the SASP could play an important role in propagated vascular inflammation and promoting lung arterial remodeling on PAH. **Table 1** summarizes the expression of endothelial senescence markers in pulmonary hypertension.

**Table 1:** Endothelial senescence markers’ expression in PH.

Endothelial Senescence Marker	Endothelial Senescence Marker detection technique- Changes	Source of sample	Reference
P53	Western Blot-I -Increased protein expression	Mice-Hypoxia-induced PH/Rat-MCT-induced PH/iPAH patients	(34)
P21	IHC- Increasing Protein Expression	COPD Patients	(35)
	qPCR and IHC- Increasing mRNA and Protein Expression	RAT- MCT+ shunt -induced PAH	(10)
	IHC- Increasing Protein Expression	COPD Patients	(29)
P16	IF- Increasing Protein Expression	iPAH patient/Mice-Hypoxia-induced PH/Mice-Sugen+Hypoxia-induced PH/Rat-MCT-induced PH	(11)
	qPCR- Increasing mRNA levels	pMVECs isolated from patients with iPAH	(10)
	IHC- Increasing Protein Expression	RAT- MCT+ shunt -induced PAH	(10)
		COPD Patients	(29)
			(35)
	scRNA-seq- Increasing gene expression	Mice-Hypoxia-induced PH/iPAH patients	(11)
CDKN2A	scRNA-seq- Increasing gene expression	PAH patient	(36)
Bcl2	Flowcytometry- High values of endothelial Bcl2 index	PH patient	(37)
MMP2	qPCR - Increasing mRNA levels	RAT- MCT+ shunt -induced PAH	(10)
IL-6	qPCR- Increasing mRNA levels.	RAT- MCT+ shunt -induced PAH	(10)
P15	scRNA-seq- Increasing gene expression	iPAH patients	(11)
Survivin	IHC- Increasing Protein Expression	PAH- CHD patient/RAT- MCT+shunt -induced PAH	(10)
SA-β-gal	SA-β-gal staining	PAH Patients	(36)

MCT, Monocrotaline; iPAH, idiopathic PAH; IHC, Immunohistochemistry; COPD, chronic obstructive pulmonary disease; qPCR, Quantitative PCR; IF, Immunofluorescence; pMVECs, Pulmonary microvascular endothelial cells; scRNA-seq, single-cell RNA sequencing; SA-β-gal, Senescence-associated beta-galactosidase; CDKN2A, cyclin dependent kinase inhibitor 2A; CHD, Congenital heart disease.

**EC senescence in PH:** One of the first reports implicating cellular senescence in PH demonstrated that impaired iron-sulfur biogenesis, resulting from frataxin deficiency, contributed to pulmonary vascular EC senescence that was associated with PH development (36). Frataxin is a mitochondrial protein and iron chaperone that plays a critical role in the formation of iron-sulfur (Fe-S) clusters (38). Reduced frataxin expression was also found to be correlated with increased p16INK4 in lung sections from patients with both PAH and PH associated with lung disease (Group 3 PH), as well as in animal models of PAH and Group 2 PH. A study using *in situ* lung specimens revealed that patients with chronic obstructive pulmonary disease (COPD) showed a higher percentage of senescent pulmonary endothelial cells by p16 and p21 staining compared to control subjects (35). Noureddine et al. (29) also reported that the percentage of senescent ECs, was higher in pulmonary vessels from patients with COPD compared to controls and positively correlated with the wall thickness area ratio (29). Wang et al. (34) demonstrated that pulmonary arterial ECs (PAECs) derived from patients with idiopathic PAH, exhibited significantly elevated expression of the senescence marker p53, which correlated with increased Bax/Bcl-2 levels compared to normal controls (34). Intriguingly, the use of a senolytic agent effectively reversed PH in these preclinical models, suggesting that targeting cellular senescence could provide a potential treatment for PH, regardless of the specific etiology (36, 39).

A recent study examined the mechanisms by which EC senescence may lead to hypoxia-induced PH using endothelial-specific progeroid mice that exhibit selectively EC aging and senescence (40). Chronic exposure to hypoxia is a well-established model that results in mild to moderate PH associated with abnormal pulmonary arterial muscularization, but not more complex arterial remodeling that is typically seen in PAH. Progeroid mice overexpress the dominant-negative form of telomeric repeat-binding factor 2 (TRF2DN) in endothelial cells under the control of the Tie2 or vascular endothelial cadherin promoter (41, 42). EC progeroid mice developed exaggerated PH in response to chronic hypoxia with greater pulmonary arterial medial remodeling compared to wild-type mice. Mechanistically, they found that progeroid mice exhibited increased endothelial expression of Notch ligands, leading to heightened Notch signaling, which promoted proliferation and migration in neighboring SMCs. Moreover, pharmacological inhibition of Notch signaling using the Gamma-Secretase inhibitor, DAPT, blocked the proliferative and pro-migratory effects of co-culture of SMCs with senescent ECs *in-vitro* and reduced PH in response to hypoxia *in-vivo* both in progeroid mice and wildtype mice. These findings support a disease-modifying role of EC senescence in PH and point to enhanced Notch signaling as potential pharmacotherapeutic target (40). However, further investigations are needed to fully define the extent of aberrant senescent-specific crosstalk that contributes to pulmonary vascular remodeling. Of note, the composition of SASP profiles varies based on the specific disease context (43). Consequently, future studies are needed to elucidate the unique SASP profile of senescent pulmonary ECs in PAH and how endothelial senescence-related signaling precisely regulates the recruitment of immune cells.

**Figure 2:**
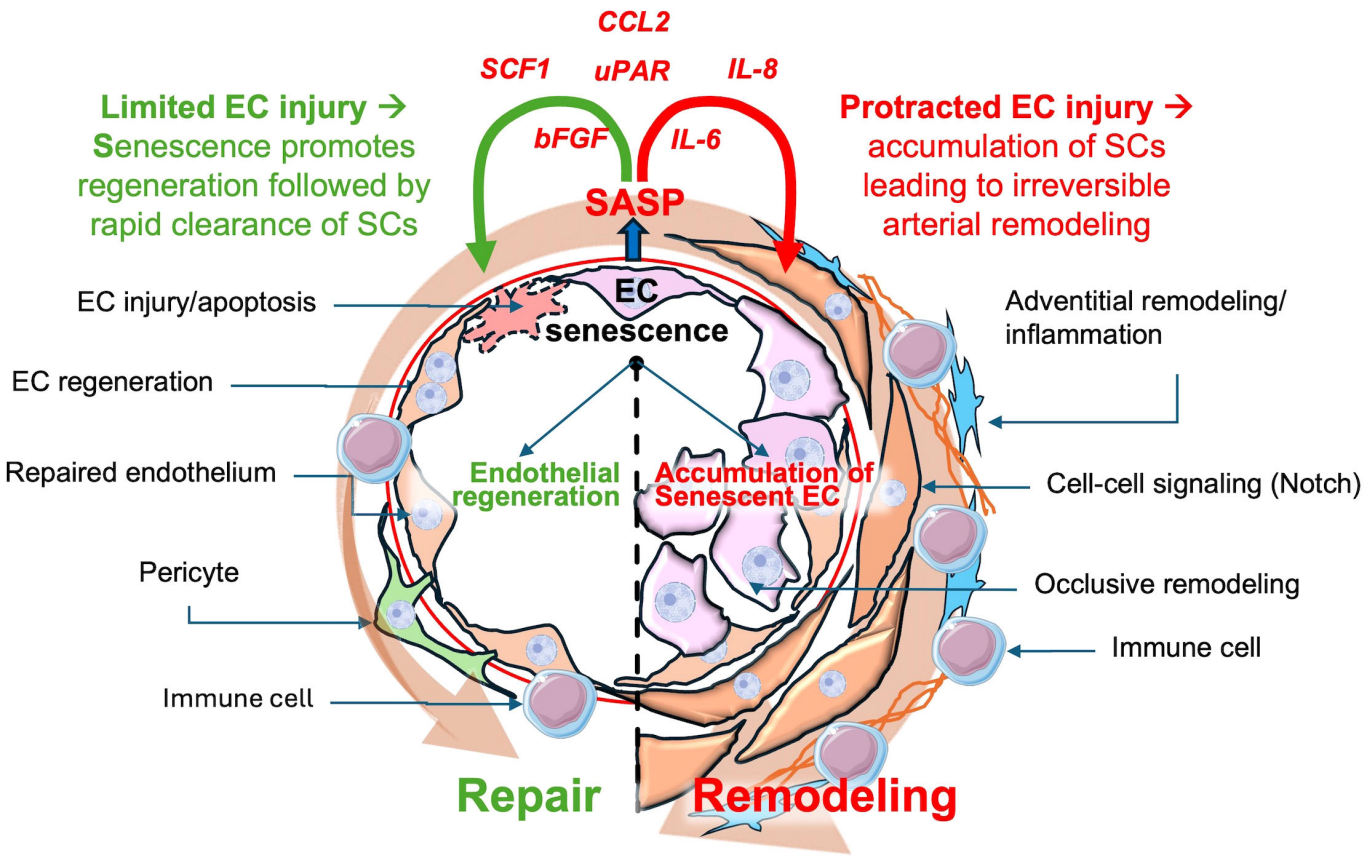
Possible dual roles of senescent endothelial cells in pulmonary arterial hypertension (PAH). Transient senescent cells in response to limited endothelial cell injury may stimulate repair, subsequently undergoing rapid clearance preventing accumulation of SCs. However, chronic endothelial cells injury can result in the accumulation of senescent endothelial cells, contributing to arterial remodeling, inflammation and PAH progression. CCL2: Chemokine (C-C motif)Ligand 2, SCF1:Stem Cell Factor 1, uPAR: Urokinase Plasminogen Activator Receptor, IL-8: Interleukin-8, IL-6: Interleukin-6, bFGF: Basic fibroblast growth factor, SASP: Senescence-Associated Secretory Phenotype.

Given the exuberant nature of the arterial pathology in PAH, which includes a profound inflammatory response, it is tempting to speculate that cellular senescence plays a key role in promoting complex arterial remodeling (**Figure 2**). While EC injury/apoptosis appears to be the key triggers for this condition, it is likely that other ‘hits’ are needed for the development of progressive disease. The study of underlying mechanisms of disease is greatly facilitated by the availability of experimental models that can faithfully reproduce its main clinical features. For many years, studies into the underlying mechanisms of PAH were limited mainly to chronic hypoxia (CH) and monocrotaline (MCT) models, neither of which fully reproduced the complex lung arterial pathology of PAH (44). CH is a model of group 3 PH due to lung disease whereas MCT is a plant-derived alkaloid that is toxic to pulmonary vascular ECs (45), but also has off target toxicity that can limit its usefulness, and again does not typically produce occlusive arterial remodeling. Perhaps the most relevant model was developed by inhibiting the VEGF receptor using a tyrosine kinase receptor antagonist, SU5416 (SU or Semaxanib). A single subcutaneous injection of SU, together with a 3-week exposure to CH, resulted in widespread lung EC apoptosis and reactive vascular cell proliferation, and a severe and progressive PAH phenotype, faithfully reproducing the hallmark lung vascular pathological features, including the complex arterial lesions (46). Recently, we have shown that perturbed blood flow, resulting in pathological levels of intimal shear stress, induces ongoing endothelial injury and apoptosis in this model, as the driver of complex and occlusive arterial remodeling (46, 47). Indeed, using unilateral pulmonary artery banding to hemodynamically offload the left lung, Abe et al. showed that ongoing hemodynamic perturbations was required for the development of complex plexiform-like lesions in an experimental model of severe PAH (46). This is analogous to the mechanism of PAH development in patients PAH associated with CHD due to large systemic to pulmonary vascular shunts, ultimately resulting in Eisenmenger’s syndrome (48, 49). In this context, excessive pulmonary blood flow causes ongoing endothelial injury, setting the stage for development of EC senescence by a variety of mechanisms implicated in endothelial dysfunction, including increased oxidative stress (15), DNA damage (50, 51) and mitochondrial dysfunction (52) (**Figure 2**). It is also possible that ongoing EC injury may overwhelm endothelial repair mechanisms, leading to proliferative exhaustion and senescent changes in regenerative cells.

## 5 Targeting cellular senescence

The importance cellular senescence in various pathological settings has made this an attractive therapeutic target for a wide range of diseases (53). Due to the absence of a precise biomarker for targeted treatment, current approaches to SC-directed therapy primarily focus on three key strategies: 1) disrupting crucial pathways that mediate senescence, such as pro-survival signaling; 2) targeting specific factors of the SASP; and (3) enhancing clearance of SCs by the immune system (20, 53).

### 5.1 Senotherapies

Senotherapies comprise an emerging class of drugs designed to eliminate SCs through promoting apoptosis (Senolytics) or altering their function (Senomorphics) (15). Currently, senolytic therapy represent the primary strategy to remove SCs *in-vivo*. Senolytic therapies involve the use of agents which inhibit cell survival pathways, and thereby induce apoptosis of SCs (**Figure 1**), some of which are in early phase clinical trials for the treatment of specific senescence-related and age-associated conditions, as well as cancer (53). However, there are concerns regarding their specificity and potential off target effects which may pose challenges to their commercial development. ABT-263 (Navitoclax) is a BCL2 inhibitor, triggering mitochondria-mediated apoptosis in senescent cells (10). ABT-263 has been evaluated in conjunction with chemotherapy in phase 1 and 2 clinical trials for different types of malignancies with an acceptable side effect profile. Additionally, other senolytics target other pathways; for example, the transcription factor FOXO4 plays a crucial role in maintaining the viability of SCs through interactions with p53, and inhibiting FOXO4 signaling could facilitate the removal of these cells and mitigate tissue homeostasis disruption caused by tissue damage or aging (54). FOXO4 forms a complex with the pro-apoptotic factor p53 thereby blocking apoptosis in senescent cells (33). To disrupt FOXO4-p53 binding, de Keizer and colleagues designed a cell-permeable peptide containing part of the p53-interaction domain in FOXO4, structured in D-retro-inverso (DRI) conformation. In senescent cells, inhibiting FOXO4-p53 binding resulted in nuclear exclusion of p53 and triggered cell-intrinsic apoptosis (33). As well, inhibitors targeting heat shock protein 90 (HSP90) have emerged as potential senolytic agents by interfering with the ability of HSP90 to stabilize proteins involved in cell survival and thereby promoting apoptosis (47). Nonetheless, a careful consideration of the context of cellular senescence is crucial in assessing whether a senotherapeutic may be beneficial or harmful in a given context, since cellular senescence can also play a key role in wound healing and tissue repair and regeneration (20). This is further complicated by the presence of different populations of SCs at the same time, as seen in recent studies demonstrating the coexistence of p16INK4a+ and p21CIP1+ SC populations in the mouse liver that have opposing effects on organ regeneration, respectively promote and impair the regeneration (55).

As well, the biological implications of cellular senescence may also depend on temporal factors; for example, early inhibition of BCL2 can interfere with repair and exacerbate pulmonary fibrosis when given during active bleomycin lung injury (56). Therefore, the lack of selectivity of current senolytic therapies represents a challenge and spatial and temporal factors must be taken into account in designing successful therapeutic strategies (20).

**Senotherapeutics in models of PAH**: The effect of senotherapies in models of PAH have been mixed and are summarized in **Tables 2**, **3**. In a rat model of CHD associated PAH induced by an aortocaval shunt together with administration of the endothelial toxin, Van der Feen et al. demonstrated the appearance of markers of SC within remodeled pulmonary arteries, including p53, p16 and p21, coinciding with the development of irreversible arterial remodeling (10). Moreover, they demonstrated that targeting cellular senescence with the BCL2 antagonist, ABT263 markedly reversed the hemodynamic and structural vascular changes of PAH, suggesting that cellular senescence was responsible for the development of irreversible arterial disease (10). Similarly, in the SU/CH rat model of PAH (12), when ABT-263 was delivered after the development of pulmonary vascular remodeling, it reduced arterial remodeling and improved pulmonary hemodynamics in both Sprague-Dawley and Fischer rat strains (12). In contrast, a recent report by Born et al. using the rat SU/CH model, as well as other rat and mouse PH models, supports a very different conclusion (11). While SCs were evident within remodeled pulmonary arteries of PAH patients and the experimental PH models, the use of pharmacological and genetic interventions targeting SCs, including activation of a conditional suicide gene driven by the p16 promoter, senolytic drugs (ABT263 and FOXO4-DRI), and inactivation of p16 in transgenic p16^LUC/LUC^ mice, significantly worsened hemodynamic and structural changes (11), suggesting that cellular senescence plays an adaptive, rather than a pathological role, in this disease.

**Table 2:** Senolytic therapies for the treatment of pulmonary hypertension.

Senolytic Drug	Mechanism of Action	Outcome	Comments
FOXO4-DRI	FOXO4-p53 interfering peptide	↑ PH/RVH	FOXO4-DRI worsened PH, causing major increases in RVSP, Fulton index, and pulmonary vessel muscularization in mice model of PH (11).
ABT-263 (Navitoclax)	BCL-2 inhibitors	↑ PH/RVH	ABT-263 increased RVSP, hypertrophy index, vessel remodeling, and decreased lung pulmonary endothelial cells in mice and rat PH models (11)
		↓ PH/RVH	ABT-263 reversed PAH in different mice and rat models (10, 12, 36)
Dasatinib	Tyrosine kinase inhibitor	↑ PH/RVH	Dasatinib treatment led to PAH development in pre-clinical and clinical studies (57–59).
17-AAG (Tanespimycin)	Heat shock protein-90 inhibitors	↓ PH/RVH	17-AAG treatment improved hemodynamic parameters, inflammation and vascular remodeling in MCT-treated rat model of PH (60, 61)
Gamitrinib			Gamitrinib treatment improved hemodynamic parameters, inflammation and vascular remodeling in MCT-treated rat model of PH (60, 61).
CB839	Glutaminase inhibitors	↓ PH/RVH	CB839 treatment improved hemodynamic parameters and vascular remodeling in MCT- treated rat model (62).
C968			C968 treatment ameliorated PH progression in MCT- treated rat model (63).

RVSP, Right ventricular systolic pressure; RV, Right ventricular; PVR, Pulmonary vascular resistance; 6MWD, 6-min walk distance; mPAP, Mean pulmonary arterial pressure; PH, Pulmonary hypertension; RVH, Right Ventricular Hypertrophy. ↓, decrease; ↑, increase.

**Table 3:** Senomorphic therapies for the treatment of pulmonary hypertension.

Senomorphic Drug	Mechanism of Action	Outcome	Data on Trials in PH
Rapamycin	mTOR inhibitors	↓ PH/RVH	Rapamycin reversed pulmonary vascular remodeling and RV hypertrophy in MCT- treated and hypoxic mice of PAH (57, 58).
		N/A	Phase I clinical trial (NCT02587325)
Everolimus (RAD001)		↓ PH/RVH	Everolimus treatment led to improvements in PVR and 6MWD in PH patients (59).
GS-444217	Apoptosis signaling kinase 1 (ASK1) inhibitor	↓ PH/RVH	GS-444217 treatment revealed dose-dependent reduction in mPAP and RV hypertrophy in MCT and Sugen-hypoxia rat models (60).
Selonsertib (GS-4997)		Neg	Completed clinical trial (NCT02234141): Selonsertib did not have any significant effect in PAH (61)
SB203580 (Adezmapimod)	p38 MAPK inhibitors	↓ PH/RVH	SB203580 treatment reduced RVSP, superoxide anion production, and inflammation in hypoxic rat model of PH (62, 63).
PH-797804			PH-797804 treatment reversed PH and decreased the production of tissue and circulating IL-6 in MCT-treated and hypoxic rat model (62).
FR167653			FR167653 treatment mitigated vascular proliferation and decreased mPAP in MCT-rat model of PH (64).
Curcumin (EF24)	Antioxidant therapy	↓ PH/RVH	Curcumin nanoparticles treatment in MCT-rat PH model was associated with reduced RV wall thickness and a decreased right ventricle weight/body weight ratio. It also reduced the upregulation of TNF-α and IL-1β mRNA expression in RV induced by MCT (65).
N-acetylcysteine (NAC)		↓ PH/RVH	NAC treatment reduced RVHI, mPAP, PVR, pulmonary inflammation score, and upregulation of ALK-1 and Smad1 in MCT- rat model (66).
		N/A	Recruiting (NCT04081012)
Apabetalone (Rvx208)	BET protein (BRD4) inhibitors	↓ PH/RVH	Apabetalone treatment reversed the PAH phenotype and improved hemodynamics parameters in different animal models of PAH (67).
		N/A	Phase I clinical trial (NCT03655704) - recruiting
JQ1		↓ PH/RVH	JQ1 reversed PAH in the Sugen-hypoxia rat model through positively influencing the balance between proliferation and apoptosis within the vascular wall (68).
Anakinra	IL-1α receptor antagonist	↓ PH/RVH	Anakinra treatment protects against PAH development in animal models and alleviated inflammation in PAH patients and RV failure who were already undergoing vasodilatory treatment (69–71).
		N/A	Phase 1 clinical trial Complete (NCT03057028)
Tocilizumab (Atlizumab)	IL-6Rα inhibitor	N/A	Recruiting (NCT04081012)
		N/A	Complete (NCT03057028)
		Neg	Phase II (NCT02676947), Tocilizumab treatment did not have any significant improvement in the PVR in PAH patients (72).
Etanercept	TNF-α inhibitor	↓ PH/RVH	Etanercept prevented and reversed PH in MCT rat model, and it decreased expression of the inflammatory cytokines in the etanercept treatment groups (73, 74).
			In Sugen-hypoxia rat model, Etanercept restored BMPR2 expression, and led to RVSP reduction, improvement of disease progression, RVH, and vascular remodeling (75).
			Etanercept prevented pulmonary hypertension in endotoxemic pig models (76).

RVSP, Right ventricular systolic pressure; RV, Right ventricular; PVR, Pulmonary vascular resistance; 6MWD, 6-min walk distance; mPAP, Mean pulmonary arterial pressure; PH, Pulmonary hypertension; RVH, Right Ventricular Hypertrophy; Neg, Negative; N/A, Not available. ↓, decrease; ↑, increase.

The apparent contradictory nature of the findings described in these reports, points to the complex biology of cellular senescence which may have dramatically divergent influences on tissue injury and repair depending on subtle differences in context. In the report of Born et al., interventions preceded or were initiated soon after the induction of PH in these models; however, when ABT263 was administered three weeks after monocrotaline-induced PH, there was a modest improvement in pulmonary arterial pressure (11). This suggests that, as in the bleomycin model of pulmonary fibrosis (56), timing of treatment with a senotherapeutic agent relative to stage of disease may be critical in determining whether this will result in salutary or deleterious outcomes. Indeed, the impressive benefits of ABT263 in the CHD model of PAH (10) occurred in the context of the delayed administration after occlusive ‘irreversible’ arterial remodeling was evident. This raises the possibility that EC senescence may not only contribute to pathological arterial remodeling, which is relevant to the late stages of disease, but it may also play an adaptive role in the initial EC response to the triggering injury, promoting endothelial repair and regeneration (**Figure 2**). Indeed, it is increasingly recognized that senescence is essential in development biology and tissue repair (20, 77), which underscores the need for nuanced use of interventions to limit the accumulation of SCs without interfering with their physiological roles in regeneration and repair.

In contrast to senolytic therapies, which induce SC apoptosis, senomorphic drugs target the factors released by SCs as part of the SASP. These compounds are often referred to as senostatics and the selection of appropriate targets requires a comprehensive understanding of the specific characteristics of the SASP in various contexts. A range of agents and antibodies have been developed to inhibit transcriptional activators of the SASP, such as NF-κB, p38MAPK, and C/EBPβ, or to neutralize specific SASP factors, such as Etanercept, Anakinra and Rapamycin (**Table 3**; **Figure 1**). In general, the effects of various senomorphic agents have been beneficial in PH and PAH preclinical models, but this has yet translated into effective therapy in early phase clinical trials. Moreover, because transcriptional regulators of the SASP also play roles in non-senescence-related functions, targeting these pathways can also result in off-target effects. Additionally, the diverse functions of specific SASP factors in different conditions, or over time, pose similar challenges in the use of senomorphics as those described above for senolytics, and pursuing a broad inhibition of the SASP may not always be advantageous (15). Finally, the systemic delivery of these agents will affect tissue and organs throughout body, which may result in unwanted side effects. One way to circumvent this limitation would be the development of inhaled therapies that would have much greater specificity for the lung.

### 5.2 Immunotherapy to enhance clearance of senescent cells

An alternative therapeutic strategy to target senescence involve the manipulation of immune cells to enhance the clearance of SCs is a rapidly evolving field of research (78–80). In healthy tissue, immune surveillance mechanisms effectively limit the accumulation of SCs (20). The failure of immune surveillance systems to efficiently recognize and clear SCs can be attributed to the development of escape mechanisms by SCs or a general decline in immune function seen in many chronic diseases (20, 81, 82). For example, levels of the MHC molecule HLA-E are increased in senescent dermal fibroblasts express in response to SASP-related cytokines, mediated by p38MAPK signaling. HLA-E interacts with the inhibitory receptor NKG2A expressed by natural killer (NK) and highly differentiated CD8+ T cells, allowing senescent fibroblasts to escape immune clearance (83). As well, aging weakens the immune system resulting in ‘immunosenescence’ and an increasing burden of SCs that can overwhelm normal immune clearance mechanisms (20), resulting in accumulation of SCs with deleterious effects on tissue function. Another example of immune evasion by senescent cells occurs in the context of cancer. Chemotherapy induces senescence in tumors, associated with upregulation of PD-L2, an immune checkpoint inhibitor (84). While not essential for senescence induction, PD-L2 enables senescent cells to evade immune surveillance, allowing them to persist within the tumor, leading to production of senescence-associated CXCL1, CXCL2, and IL6, which are involved in the recruitment of myeloid-derived suppressor cells (MDSC). In contrast, PD-L2 blockade synergizes with chemotherapy, inducing remission in mammary tumors in mice (84). In another study, Muñoz et al. identified that senescent cells can evade immune recognition through matrix metalloproteinase (MMP)-dependent shedding of NKG2D ligands, reinforced by paracrine suppression of NKG2D receptor-mediated immunosurveillance (85).

The precise identity of the endogenous immune surveillance mechanisms mainly responsible for mediating clearance of SCs *in-vivo* remains unclear. A variety of different immune cell types have been implicated in clearance of SCs, including macrophages, NK cells and cytotoxic T cells. SC phenotypes vary depending on the specific disease and triggering factors, and it is evident that various types of immune cells can collaborate in the elimination SCs (78). Surface markers, such as Dipeptidyl peptidase-4 (DPP4), expressed on many types of senescent cells, including SCs (86), can serve as potential targets for NK cells (20). Administration of anti-DPP4 antibodies can potentiate NK cell-mediated destruction SCs (87) as demonstrated by Kim et al. who reported that SCs can be selectively eliminated via antibody-dependent cell-mediated cytotoxicity (ADCC). This phenomenon is attributed to the susceptibility of SCs, owing to the presence of DPP4 on their surface, to destruction by NK cells recognizing anti-DPP4 antibodies. Another study has demonstrated that NK cells play a critical role in surveillance of senescent activated stellate cells within the liver. Senescence-activated hepatic stellate cells upregulate MHC class I polypeptide–related sequence A (MICA) and UL16 binding protein 2 (ULBP2), which are ligands that activate NKG2D receptor of NK cells (79). NK cells have been shown to play a critical role in surveillance of senescent activated stellate cells within the liver (79, 88). For example, deletion of the NKG2D receptor led to the accumulation of senescent stellate cells, culminating in elevated liver fibrosis levels in mice (88). In contrast, activation of NK cells through polyinosinic-polycytidylic acid decreased the SCs and led to resolution of liver fibrosis (54, 81). Moreover, recent reports have demonstrated that the cytotoxic effector perforin-granzyme pathway is essential for the eliminating SCs in various tissues during the aging process and in cases of chemically induced liver fibrosis (89, 90).

The recent advances in immunotherapeutic strategies to target cancer cells may provide novel tools for the clearance of disease associated SCs. Specifically, a chimeric antigen receptor (CAR) targeting urokinase-type plasminogen activator receptor (uPAR), a cell-surface protein found on oncogene-induced SCs was used to target surface markers of senescence (47). uPAR-specific CAR T cells demonstrated remarkable efficiency in eliminating SCs in lung adenocarcinoma and liver fibrosis models *in-vivo* (91). This approach is particularly well-suited when SCs express an abnormal antigen within a specific tissue context, such as in oncogene-induced senescence linked to cancer, as opposed to chronic diseases associated with DNA damage-induced senescence. To overcome the need for antigen specificity, Arora et al. used invariant NKT (iNKT) cells to enhance SCs clearance (81). They showed that the administration of glycolipid antigens (i.e., α-GalCer) resulted in activation and amplification of iNKT cells, thereby inducing an endogenous surveillance system for removal SCs in disease models. They used two distinct disease models associated with senescence, chronic high-fat diet (HFD)-fed mice model of adipocytes senesence and bleomycin-induced lung injury model of interstitial lung fibrosis. Activation of iNKT cells by a-GalCer, or adoptively transferred iNKT cells, was sufficient to result in effective senescent cell elimination *in-vivo*, resulting in improved disease outcomes (81). These findings highlight the potential of tissue-resident iNKT cells to enhance SC clearance and lays the foundation for anti-senescence therapies that target these cells and the mechanisms driving their activation. Therefore, senescence immunotherapy holds significant promise as an alternative to senolytics and senomorphic therapies for enhancing SC clearance in the context of aging and chronic diseases. Various immune cells possess specific abilities to detect and eliminate distinct senescent cell types, underscoring the importance of characterizing these cells in different disease contexts for the development of advanced strategies.

## 6 Conclusion

There is great enthusiasm for the development of treatments that target senescent cells across a wide variety of diseases. However, the lack of a universal senescence biomarker poses challenges for translational efforts, necessitating detailed characterization of SCs in different disease contexts. Determining when and how to target SCs is crucial due to the pleomorphic and dynamic nature of biological effects on cellular senescence, requiring in depth understanding of senescence responses in specific settings to identify optimal therapeutic strategies and windows, highlighting the need for comprehensive phenotypic characterization to inform treatment design. The role of EC senescence in PAH is still poorly defined, necessitating careful assessment of the biology of cellular senescence in preclinical models to determine optimal treatment strategies and timing. Conflicting outcomes with the use of senolytic therapies in preclinical models of PAH necessitate further investigations to identify the specific characteristics of senescent ECs and define the spatial and temporal factors that determine their biological effects during disease evolution. Utilizing advanced technologies, such as single-cell transcriptomics and proteomics, will be instrumental in identifying which specific cells undergo senescence in PAH and defining at high resolution the spatiotemporal sequences of senescent changes during disease evolution. This detailed cellular and mapping could inform the development of more effective senotherapies by allowing for more precise targeting of senescent cells. As well, more tailored strategies, such as cell-type-specific or targeted immunotherapeutic approaches, will be essential in the design of the next generation treatments.

## Author contributions

ESQ: Writing – original draft. DS: Writing – review & editing.
